# INCREASING TECHNOLOGY ACCESS TO FOR UNDERSERVED COMMUNITIES: CATCH-ON CONNECT

**DOI:** 10.1093/geroni/igac059.550

**Published:** 2022-12-20

**Authors:** Erin Emery-Tiburcio, Siqi Wang, Steven Reaves, Salvador Castaneda

**Affiliations:** Rush University Medical College, Chicago, Illinois, United States; Rush University Medical Center, Chicago, Illinois, United States; Rush University Medical Center, Chicago, Illinois, United States; Rush University Medical Center, Chicago, Illinois, United States

## Abstract

Many older adults from underserved communities do not have access to technology or a reliable internet connection. Even when access is not an issue, older adults often find technology challenging. CATCH-ON Connect provides community-dwelling adults age 65+ with cellular-enabled tablets with pre-installed apps to enable them to reduce social isolation and engage in telehealth visits. This program also provides personalized technical assistance and education about accessing electronic health records and the 4Ms. For example, participants are encouraged to address the 4Ms during telehealth visits. Among 139 participants enrolled, 51% were non-Hispanic Black, 30% non-Hispanic white, 13% Hispanic, and 7% other. Willingness to engage in telehealth appointments increased 9% (p<.05), discussions with primary care providers about mobility increased by 13% (p<.001), and loneliness scores decreased by 0.6 (p<.05) in the first three months of participation. Implementation and policy implications for ongoing technology device and training services will be discussed.

